# The Underlying Molecular and Network Level Mechanisms in the Evolution of Robustness in Gene Regulatory Networks

**DOI:** 10.1371/journal.pcbi.1002865

**Published:** 2013-01-03

**Authors:** Mario Pujato, Thomas MacCarthy, Andras Fiser, Aviv Bergman

**Affiliations:** 1 Department of Systems and Computational Biology, Albert Einstein College of Medicine, Bronx, New York, United States of America; 2 Department of Biochemistry, Albert Einstein College of Medicine, Bronx, New York, United States of America; 3 Department of Applied Mathematics and Statistics, SUNY, Stony Brook, New York, United States of America; Insitute for Systems Biology, United States of America

## Abstract

Gene regulatory networks show robustness to perturbations. Previous works identified robustness as an emergent property of gene network evolution but the underlying molecular mechanisms are poorly understood. We used a multi-tier modeling approach that integrates molecular sequence and structure information with network architecture and population dynamics. Structural models of transcription factor-DNA complexes are used to estimate relative binding specificities. In this model, mutations in the DNA cause changes on two levels: (a) at the sequence level in individual binding sites (modulating binding specificity), and (b) at the network level (creating and destroying binding sites). We used this model to dissect the underlying mechanisms responsible for the evolution of robustness in gene regulatory networks. Results suggest that in sparse architectures (represented by short promoters), a mixture of local-sequence and network-architecture level changes are exploited. At the local-sequence level, robustness evolves by decreasing the probabilities of both the destruction of existent and generation of new binding sites. Meanwhile, in highly interconnected architectures (represented by long promoters), robustness evolves almost entirely via network level changes, deleting and creating binding sites that modify the network architecture.

## Author Summary

Development from egg to embryo depends to a large extent on regulatory networks of genes called transcription factors. Previous research has shown these gene regulatory networks to be robust to perturbations at the level of the connections between transcription factors. Here, we investigate the mechanisms underlying the evolution of robustness in gene networks using a modeling approach, which considers three levels: binding of individual transcription factors to DNA, dynamics of gene expression levels, and fitness effects at the population level. In our model the gene regulatory network is determined by transcription factor binding sites within DNA sequences, which undergo mutation. We categorize these mutations in a continuum ranging from silent mutations, which have no effect on regulation and change only the DNA sequence (local-sequence level), to mutations that change connections between genes in the network (network-architecture level). We find that in sparse networks, containing few connections between genes, a balance of local-sequence and network-architecture level mechanisms are responsible for the evolution of robustness, but when the network is densely connected the network-architecture level mechanisms become dominant. We argue that the shift towards the network-architecture level for more densely-connected networks offers a potential explanation for the evolution of increased complexity.

## Introduction

Robustness to genetic and environmental perturbations is ubiquitous in biological systems [1]. An abundance of theoretical and experimental evidence has shown robustness operating at many levels ranging from microRNA precursors [2] to metabolic pathways [3] and gene regulatory networks [4]. By definition, genetic robustness will facilitate the accumulation of genetic variation in a population, which in turn may prove useful for adaptation or evolvability [5]–[7]. Previous theoretical studies using gene regulatory network models have shown how robustness evolves under conditions of stabilizing selection [8]. In this class of models, mutations were allowed to alter only the interaction strengths, constraining the model to fixed network architectures. Therefore these models were not suitable to explore potentially important factors such as evolved redundancy [9], modularity [10] and degeneracy [11], [12]. Model refinements that allowed the network architecture itself to evolve have highlighted the importance of network lability even under conditions of stabilizing selection, while making arbitrary and sometimes conflicting assumptions about gain and loss of interactions [13], [14]. The network lability seen in these cases coincides with genomic studies where, for example, high rates of gain and loss of *cis*-regulatory elements are observed [15]–[17] including cases where function is highly conserved [18].

The actual mechanisms underlying changes in network interactions will predominantly involve mutations at the sequence level in *cis*-regulatory regions [19], [20] rather than changes in protein sequence and structure [21]. Even though many transcription factors binding sites (TFBSs) have been characterized in detail [22], [23], there is still a limited understanding of the evolutionary forces involved in the creation and maintenance of these TFBSs in the context of a gene regulatory network. Relevant theoretical studies have addressed evolution of *cis*-regulation by considering single TFBSs or groups of TFBSs within a single *cis*-regulatory region, for example, by calculating the distribution of canonical site variants under mutation-selection balance [24], [25]. One potential mechanism for achieving robustness at the sequence level is TFBS redundancy [26], [27], i.e., the maintenance of multiple copies of binding sites for a particular transcription factor. A variety of evolutionary forces may be involved in maintaining TFBS redundancy, including recombination within the *cis*-regulatory region or simply the length of the promoter region, which increases the probability of creating a binding sites *de novo* [9].

The evolution of robustness in gene regulatory networks is likely to involve mechanisms at both the local-sequence and network-architecture levels. Until now these two levels have been considered separately. Here we present a model that combines these two levels, enabling us to address their relative influence and how they interact in the context of the evolution of robustness. At the sequence level we use structural models of transcription factor(TF)-DNA interactions to estimate binding specificities for all possible DNA binding sites, which allows an explicit sequence-level representation of upstream regulatory regions (URRs) in determining the architecture of the gene regulatory network. Point mutations drive changes at the sequence level of individual TFBSs (which can change the binding specificity) or at the network-architecture level by creating or deleting interactions. Using this model we are able to quantify the relative contributions of the sequence and network level mechanisms to the evolution of robustness. We find that in sparse architectures, reflected in the use of short URRs, a mixture of local-sequence and network-architecture level changes are exploited, whereas in highly interconnected architectures (simulated with long URRs) the balance shifts almost entirely to the network level.

## Results

### Molecular model of gene regulatory networks

The model is implemented on three levels: (1) TF-DNA interactions, (2) gene expression and (3) population dynamics. On the first level, TF-DNA interaction strengths for all possible DNA sequences of 8 base pairs in length (8-mers) are obtained from experimentally solved structures of protein-DNA complexes. At the gene expression level, each gene is regulated via explicit promoter sequences or URRs, which are scanned for binding sites to build up a matrix of interaction strengths between all transcription factors and all promoter regions, to determine the gene expression dynamics within the context of a standard network model. At the population level, genotypes (defined in terms of explicit URRs) undergo cycles of reproduction, mutation and selection. We now describe these levels in detail.

(1) TF-DNA interactions: as described in He *et al.* [28], statistical weights, *q_x_* can be associated with individual DNA sites, *x*, upon a TF binding as:
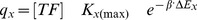
(I)where [*TF*] is the concentration of TF, *K_x(max)_* is the binding affinity of the consensus site (lowest energy site), *β* the Boltzmann constant and *ΔE_x_* the binding energy difference of site *x* relative to the consensus site *x(max)*. Binding energies of a TF with all possible *x* sites are difficult to assess experimentally. Previous studies have used a variety of methods to approximate these TF binding energies [28]–[31]. For example, computational methods have used known DNA position weight matrices and assumed additive interactions between DNA bases [28], [31]. Protein binding microarray technology [22] offers an experimental approach to measure binding strength of TFs, but the compression of all possible 10-mers in the array produces a convoluted signal that is not trivial to decode. The proper solution to this problem remains under debate [32], [33]. Alternatively, we use a computational method to obtain TF binding preferences based on structural information from TF-DNA complexes. Our approach uses a similar framework to that of Morozov *et al.* [29]. The main difference is that we use an atomistic statistical pair potential function to estimate TF-*x* interactions (where *x* is an 8-mer site) [34] instead of the Rosetta potential [35]. Similarly, we obtain a statistical score *ε_x_* for each site *x*, that represents an estimate of the binding strength between TF and *x*. To be comparable, *ε* scores must be normalized, since they scale linearly with the number of atomic contacts at the TF-DNA binding interface (Fig. S1). A convenient way to achieve this [31] is to reference them to the consensus sequence, *x(max)*, as follows:
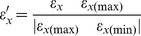
(II)where *ε_x(min)_* represents the *ε* score of the least favored binding site.

**Figure 1:**
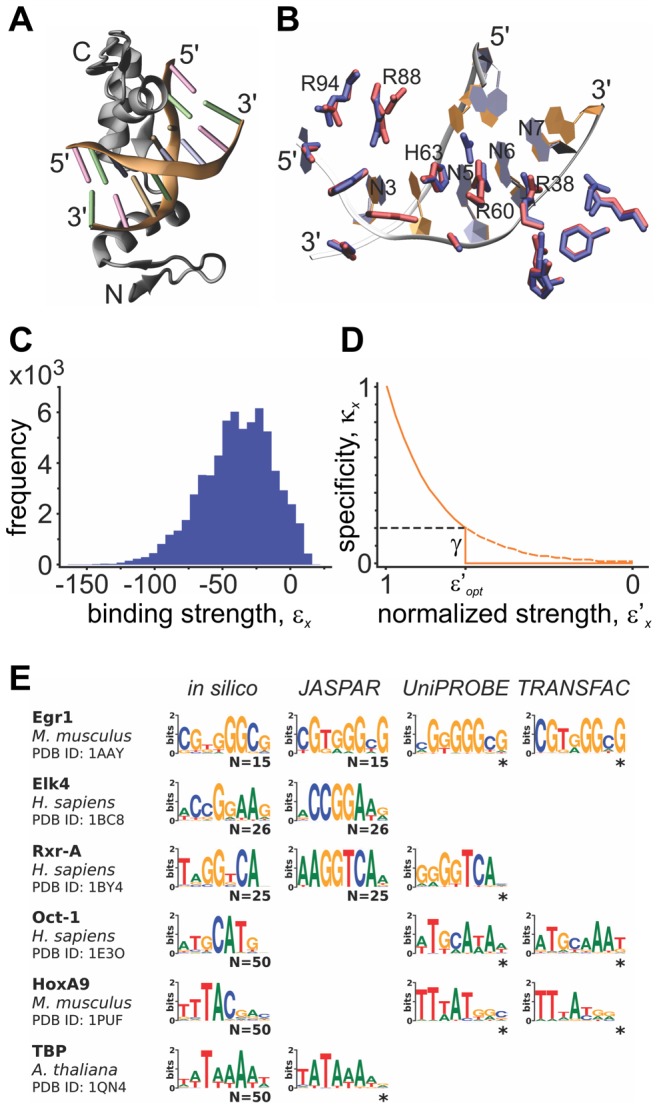
Determination of transcription factor binding sites and relative binding specificities by *in-silico* molecular modeling. (A) Example of *in-silico* model of DNA-protein complex for the transcription factor EGR1 (PDB:1AAY, originally with sequence 5′-GCGTGGGC-3′) bound to the candidate 8-mer 5′-CGTTGTCG-3′. DNA color codes: GUA:green, CYT:pink, ADE:blue, THY:orange. (B) Detailed view of same model complex for protein residues at 3.5 Å distance from DNA, showing residue repositioning upon energy minimization procedure. Here, the crystal structure is shown in blue and the model in red. (C) Distribution of calculated binding strengths, *ε*, using the Robertson-Varani statistical potential on TF-DNA complexes for all possible 8-mers (4^8^) for the Egr1 structure. (D) Transformation of normalized *ε* scores into relative binding specificities, *κ*. Dashed line indicates cutoff level *γ*, below which all specificities are set to zero, providing a variable separation between binding and non-binding 8-mers. *ε′_opt_* is a particular value of *ε′*, defining constant numbers of binding sites for each TF (see Materials and Methods). (E) Six *in-silico* determined TFBS preferences were compared against those available in JASPAR [23], UniProbe [35] and TRANSFAC [36] databases. *N* indicates the number of sequences used (we used the *N* lowest energy sequences to obtain *in-silico* preferences) to produce the information-content sequence logos (WebLogo [60]). *Logos constructed from frequency matrices.

Analogously to eq. I, we can calculate statistical weights for binding as:
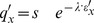
(III)where *s* is the relative concentration of TF (on a scale from 0 to 1), *ε′_x_* is the normalized statistical score of *TF-x* interaction (with *ε_x(max)_ = *0 for the preferred site) and *λ* a scaling factor. *λ* controls the slope of the exponential and is a free parameter of the model. The term 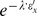 is analogous to a binding affinity or specificity (referred to as *κ_x_* from now on). It is displayed as a function of *ε′_x_* in Fig. 1D and is nearly zero for the majority of the putative binding sites. Similarly, *κ_x(max)_* is analogous to *K_x(max)_* from eq. I and becomes 1 after referencing the binding strengths to the consensus site, *x(max)*. We further applied a cutoff *γ* on the specificity *κ_x_*, to differentiate specific from non-specific TF binding, thus defining the set of TFBSs. Different cutoffs are explored by varying the parameter *γ*. The values of *γ* and *λ* were chosen to generate the expected number of TFBSs within the range 60 to 900, as previously estimated for a wide range of transcription factors in mouse using protein binding microarrays [22]. We identified *ε′_opt_* = 0.209 (see Fig. 1D), as an optimal value of *ε′_x_* to obtain the expected number of TFBSs closest to the average in the range of 60–900 for all studied TFs in the model (Table S1 lists the specific number of TFBSs used for each TF). *γ* and *λ* are linked in eq. III through the value of *ε′_opt_* (
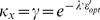
). Changes to the slope of the exponential are accompanied by a change in the specificity cutoff, γ, thus modulating the relative differences in specificity between binders. Various values of *γ*, which we will refer to as ‘specificity gap’ from here on, were explored (0.05, 0.10 and 0.20) representing an increasing discrimination between specific and non-specific TFBSs.

We compared the predicted binding site preferences that we obtained from our above described *in silico* approach with experimentally-determined preferences from the JASPAR [23], UniProbe [35] and TRANSFAC [36] databases for a subset of 6 TFs for which literature data is available, (Fig. 1E). For most cases the computationally determined binding site preferences are very similar to the experimentally-determined preferences. The only exception is the transcription factor Rxr-A, for which none of the known or computationally calculated motifs agree in the first two positions. The overall good agreement demonstrates the usefulness of our scoring procedures to recapitulate binding preferences.

We now describe in more detail how we acquire *TF-x* interactions from structural models. A set of 10 TF-DNA crystal structure complexes were chosen from the Protein Data Bank (PDB) [37]. For each complex, we exchanged the DNA bases in all possible combinations (limiting the length of the binding site to 8-mers) using the NAMD 2.6 package [38]. In this way, we generated 4^8^ TF-DNA complexes (see Materials and Methods for further details). Using the same software, we optimized TF-DNA atomic interactions with an energy-minimization procedure. The atomistic statistical pair potential method described above, based on pairwise TF-DNA contacts, was used to obtain a score, *ε*. Fig. 1A shows the modeled structure of the Egr1 transcription factor bound to the 8-mer 5′-CGTTGTCG-3′, based on the Egr1-DNA crystal structure (PDB code: 1AAY). This particular 8-mer ranked 300th (*ε_x(300)_* = −116.04) from the best scoring complex (*ε_x(max)_* = −163.12), which corresponds to the original crystal structure bound to the consensus sequence, 5′-GCGTGGGC-3′. Fig. 1B illustrates the conformational differences of protein side-chains, located at the interface (residues located within 3.5 Å of DNA atoms), in the modeled structure (in red) when compared to the crystal structure (in blue). These atomic rearrangements (typically displaying an RMSD difference of less than 0.5 Angstrom) are the result of the employed energy-minimization procedure, which relaxes the molecular interactions in the complex to accommodate the new DNA sequence. The largest changes are observed, as expected, on residues contacting specific DNA bases, for example R88 that makes specific contacts with base N3. The set of *ε* scores for the Egr1 factor displays a Boltzmann-like distribution, where only a small fraction of sequences are recognized with favorable scores (Fig. 1C), similar to observations made for various stable structural features in globular proteins [39], [40].

Previous works on this topic often employed Position Weight Matrices (PWM) to estimate binding affinity of TF-DNA interactions. The reason why we decided to employ the above described more elaborate approach is because the PWM model assumes independency of interactions with each base in the DNA and therefore the accuracy of this model has been questioned extensively in the literature [41], [42].

**Figure 2:**
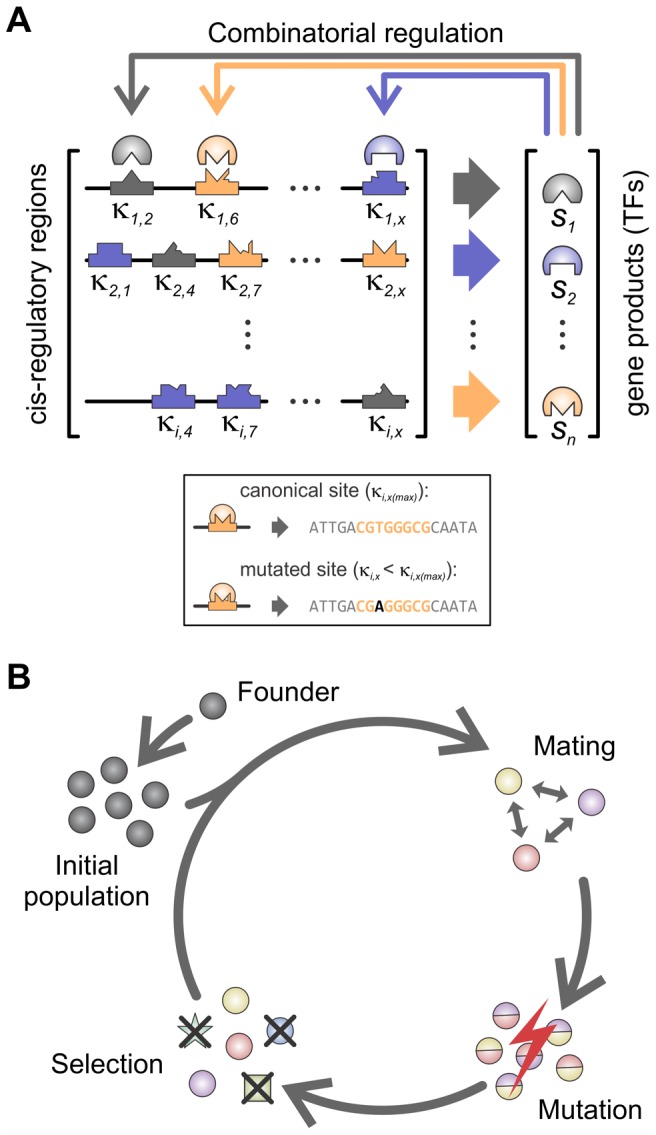
Schematic representation of the gene-regulatory network model. (A) Model of development. The expression of each gene is regulated by combinatorial interaction between an explicitly modeled *cis*-regulatory sequence (black lines) and the gene products (sequence specific transcription factors). Each gene product is represented by a different color. Shapes within the *cis*-regulatory regions represent sequence determinants of regulatory elements and their colors define the identity of the interacting transcription factor. Within the box, the explicit regulatory sequence representation is illustrated by showing an example of a consensus binding site for a given TF (maximal binding specificity, *κ_max_*) and a mutated site (with a lower *κ*). The extent of gene regulation is a function of the presence and associated binding specificities of each regulatory element (*κ_ix_*, where *i* is the input gene and *x* is a regulatory site on gene *j*), transcription factor abundances (*s_i_*) and the function of the interacting transcription factors (activator or repressor of transcription, represented as positive and negative *s_i_* values). (B) Population model. Simulations start with a randomly chosen developmentally stable founder. Variation is introduced in two forms: exchange of promoter regions between two randomly chosen parents (without recombination within promoter regions) and single point mutations at the DNA level. Selective pressure is applied to the offspring on two levels: they must develop a stable expression pattern through time (phenotype) and that phenotype must be similar to that of the founder.

(2) Gene expression: We use the TFBSs to define a gene regulatory network model (Fig. 2A) using a set of 10 transcription factors (Table S1). We assign to each TF gene an URR of length *L* (values of *L* = 50, 100, 200 and 300 bp were explored). Initially, the URR is chosen to be a random DNA sequence with equal probability occurrences of G, C, A or T bases. The total number of overlapping 8-mers in each URR is *L-7*, although only a fraction of these will be actual TFBSs (as determined by the value of *x*). To build a matrix *w* of interactions between each TF *j* and a regulated gene *i*, we scan the URR of each gene *i* for TFBSs of gene *j* (including overlapping TFBSs) and assign to each *w_ij_* entry the sum of *κ_x_* values. Promoter length in our model determines the number of connections between genes, also known as network connectivity, which is defined as the fraction of nonzero entries in the matrix *w*. Each gene *j* is randomly assigned a role as either a transcriptional activator (+1) or repressor (−1) via the sign of *v_j_*, which remains unchanged throughout the simulation. Similarly to previous models [8], [43], [44], we define expression levels 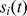 for each gene *i* over time *t* as:
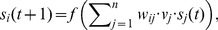
(IV)where 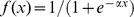 is a sigmoid function with steepness *α*. The initial state, 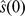, is constant for each simulation and is set by randomly choosing each 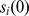 to be 1 (on) or 0 (off). The equation is iterated until either the system reaches a steady state expression 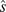, or a time limit (see Materials and Methods). Only those individuals that reached a steady state are considered viable.

(3) Population dynamics: Here, we largely follow previous models [8], [43], [44] with the exception of the mutation operation (Fig. 2B). Initially, a random individual (the Founder) is generated and required to be viable, where its steady state output is defined as the optimal phenotype, 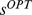. This Founder is then cloned to form an initial population of size *M* = 500. Subsequent generations are produced via cycles of reproduction, mutation and selection. For reproduction, offspring for the next generation are created via sexual reproduction. Pairs of individuals from the parental population are chosen at random, then random URRs are picked from either parent and inserted as the corresponding URR of the offspring (parental pairs are sampled with replacement from the population). Mutation of the URRs is implemented by randomly replacing base pairs at a fixed rate of 1 mutation per 100 bp of DNA per genome. Selection has two components: first, viability such that the offspring are required to reach steady state (typically, less than 5% of the offspring need to be replaced at each generation), and second, a fitness measure based on how close the phenotype 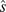 is to that of the founder, 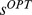 (see Materials and Methods). Offspring are generated and undergo selection until a new population of size *M* is reached.

### Robustness increases with increasing promoter length and specificity gap

Genetic robustness is defined as the difference in phenotypes between a perturbed and an unperturbed individual [8], [45]. A smaller phenotypic difference means that the individual has greater tolerance to perturbations and is therefore more robust. Here, we measured genetic robustness by testing the phenotypic consequences of single point mutations inserted into the URRs of TF genes in the network. Previous investigations using similar network models have shown the evolution of genetic robustness [8], [43], [44]. Phenotypic differences or effects, monitored by the Euclidean distance between perturbed and unperturbed phenotypes, emerge as a combination of two components, due to stable individuals (those that develop a stable phenotype after being perturbed) and to unstable individuals (those resulting in an unstable phenotype). In all simulations, stable individuals completely dominate the phenotypic effect (above 97% of the perturbed individuals develop a stable phenotype). In this work we focus on robustness coming from the fraction of stable individuals because the contribution of unstable individuals is negligible and the measured phenotypic distances of unstable individuals adopt large and random values that are uninformative for the purposes of this work (Fig. S2).

**Figure 3:**
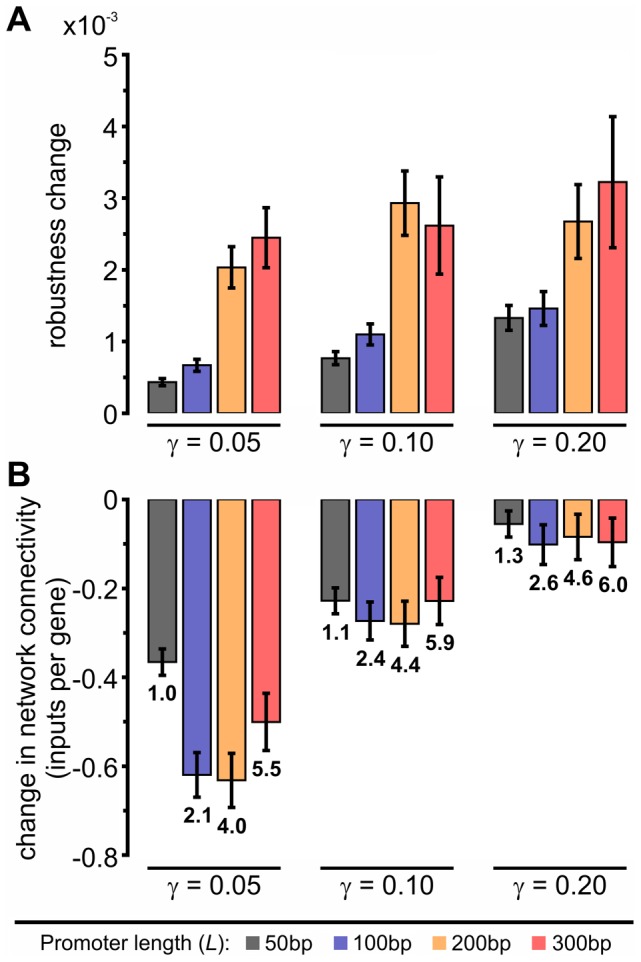
Evolution of robustness depends on URR length and specificity gap. (A) Change in robustness, measured as the difference of the mean phenotypic distances between unperturbed and perturbed individuals at generations 2000 and 0. The mutation rate used for this measure was 1 mutation per 100 bp per genome. (B) Change in connectivity (comparing generation 2000 to generation 0), measured by the fraction of unique inputs in the network of a given individual. The numbers at the end of each bar represent the connectivity at the end of the simulations. Error bars are the standard error of the mean over 100 independent simulations.

Using our model, we performed experiments consisting of 100 independent simulations under strong selection (see Materials and Methods), each lasting 2000 generations with robustness evaluated every 10 generations. We systematically varied the two different free parameters of the model: the length of URRs, *L*, and the specificity gap, *γ* (Fig. 1D). In Fig. 3A we show the robustness change, defined as the inverse of the difference between the final and initial phenotypic distances (generations 2000 and 0, respectively), as a function of *L* and *γ*. Robustness change is computed for each individual and averaged over the entire population. By generation 2000, robustness levels out, reaching a maximum value in every case (Fig. S3, upper panel). To confirm that robustness saturated, we calculated p-values for the entire length of the simulations in reference to the level of robustness at generation 2000, using t-test (Fig. S3, lower panel). Consistent with previous results using fixed-architecture models [8], [43], [44], we found that the robustness change increases as a function of URR length or network connectivity (observed in any subset of *γ* values in Fig. 3A).

Variations in *γ*, the specificity gap between binders and background sequences, also show an effect in the change in robustness. Fig. 3A shows that our model networks achieve greater robustness at larger values of *γ*. We could interpret the specificity gap, *γ*, as a conformational change upon binding in any of the parts in the TF-DNA complex (not explicitly modeled), potentially producing an abrupt change in binding specificity. In the literature there are many well-known examples of this phenomenon, such as the dramatic bends produced at specific DNA sites by the TATA binding protein [46] and LacI repressor [47]. Other cases include folding coupled to binding in different families of TFs, like helix-turn-helix motif containing cytidine repressor [48]) and bZIP (Jun and Fos) [49]. Also, large changes in side chain conformers at the binding interface have been reported, for instance in the case of PhoB [50]. In summary, conformational changes upon binding of TFs to DNA are recognized to be the rule rather than the exception [51]. This suggests that nature has evolved a rather discrete discrimination (specificity gaps) between specific and non-specific binding as an alternative to smooth specificity transitions.

Another important parameter in our model is network connectivity. Mutations in the DNA sequence modulate the quantity as well as the specificity, *κ*, of TFBSs, thus producing natural rates of deletion and creation of TFBSs. The initial network connectivity is controlled by the length of the URRs (*L*) (Fig. S4). The changes in connectivity in our simulations are shown in Fig. 3B, where the values of the final connectivity marked at the end of each bar. We observe that connectivity drops in every scenario (more dramatically at lower *γ*). This observation in general is consistent with other studies that used numerical models, where the fixed-architecture constraint was relaxed using rates of deletion or creation of connections. It was suggested that lowering the connectivity presents less opportunities for disruptive mutations, which in turn can generate a force towards sparser architectures [13]. However, in our studies under conditions of high values of *γ*, the network connectivity remains unchanged, showing that the final network connectivity depends only on the specificity gap, *γ*, and not on the URR length. Our results at lower *γ*, show that the system reduces connectivity by eliminating TFBSs with very low *κ* values, probably because these have negligible effect on protein expression and therefore do not impact the phenotype of the individuals. The observations about the role of *γ* highlight the importance of including molecular details in the model.

### Local-sequence level mechanisms drive robustness in systems with shorter promoter lengths

**Figure 4:**
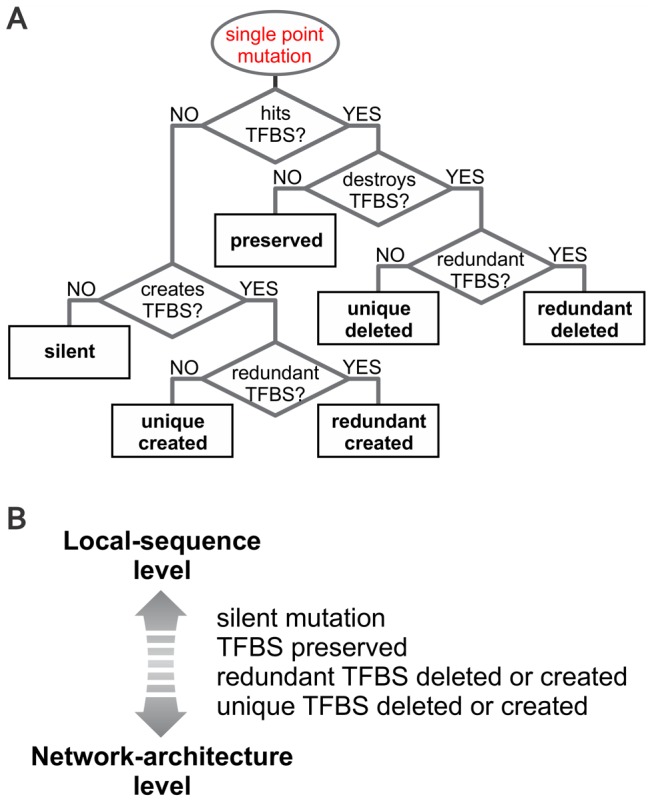
Classification of events produced by single point mutations on a *cis*-regulatory segment. (A) Decision tree defining all possible events on *cis*-regulatory regions after the introduction of a point mutation. (B) These events can be thought of as “tools” available to the system, since they summarize all the changes the system can potentially make. For clarity, we also classified them in a continuum, according to their impact on the network architecture. Silent mutations are located at the local-sequence level extreme, since they produce changes that only affect the sequence without modifying either network architecture or gene expression levels. On the other hand, deletion or creation of unique TFBSs is found at the other extreme (network-architecture level) because these events directly impact the network's architecture. A preserved TFBS has the ability to change the relative specificity of a binding site.

To assess the underlying mechanisms that contribute to the evolving robustness, we proceed by categorizing each mutation in a URR sequence. Since we know the positions of each TFBS in a given URR, we can identify whether a mutation affects a TFBS or not. If the mutation affects a TFBS, we can further distinguish whether the TFBS is destroyed as a result of it and if so, whether it represents a unique (non-redundant) input for a gene (see decision tree in Fig. 4A). Similar distinctions can be made for cases where the mutations avoid TFBSs. Although a single mutation can trigger multiple events (e.g. a deletion and simultaneous creation of a TFBS from a different TF) we observed that the relative frequencies of the components within a multiple event mirror those of the single events (Fig. S5). We will therefore focus on the contributions of the single-event categories.

Each of the mutation types (Fig. 4A) can also be categorized on broader conceptual levels (Fig. 4B) between two extremes: local-sequence and network-architecture level changes. At the extreme of network-architecture level, mutations qualitatively change the network architecture either by adding an interaction between two previously unconnected genes (“unique created”), or by deleting a non-redundant link between two genes (“unique deleted”). At the extreme of local-sequence level, the network architecture is unaffected (“silent mutations”). Mutation types that change interactions quantitatively fall between these two extremes.

**Figure 5:**
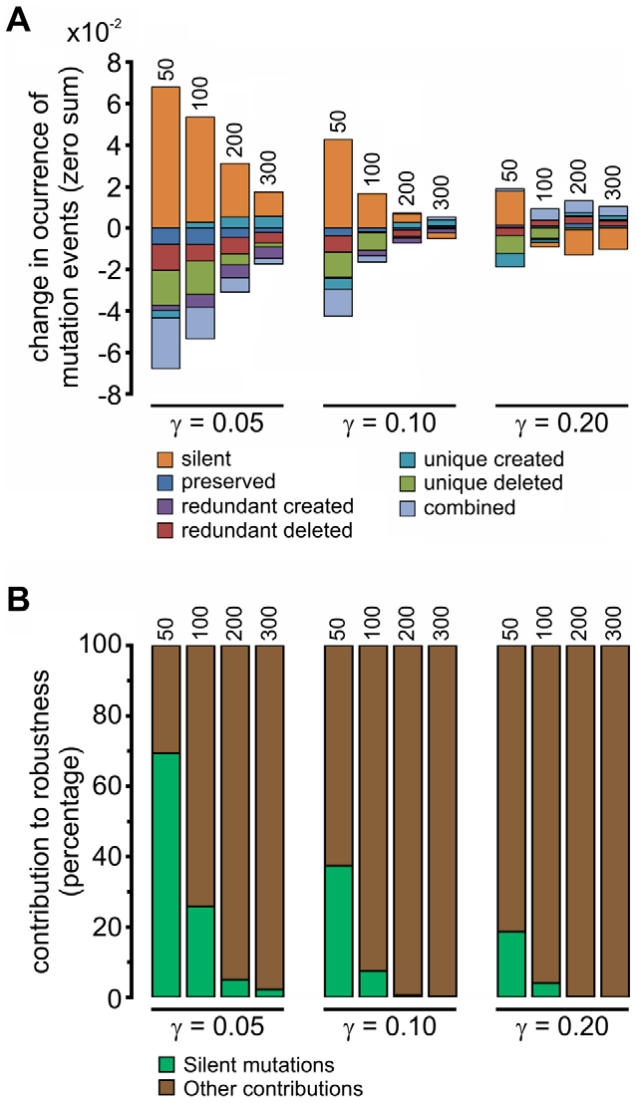
Decomposition of robustness. Robustness due to stable individuals is the sum of the products between the frequency and the average phenotypic distance of the mutational events described in Fig. 4A. Therefore they can be used to decompose robustness. (A) Relative composition of the frequencies of each mutation type. They were measured as the differences between final and initial generations in the simulations for each of the classified mutational events (see Materials and Methods). Silent mutations dominate in almost all cases, especially at low specificity gap (*γ*) and URR lengths (*L*). Silent mutations are found at the extreme of local-sequence level changes (Fig. 4B). (B) Fraction of local-sequence and network-architecture level changes. Local changes were calculated as the fraction of the total robustness change assuming constant frequency of silent mutations (see Materials and Methods). The length of the URRs (in base pairs) is indicated on top of each bar in both graphs.

**Figure 6:**
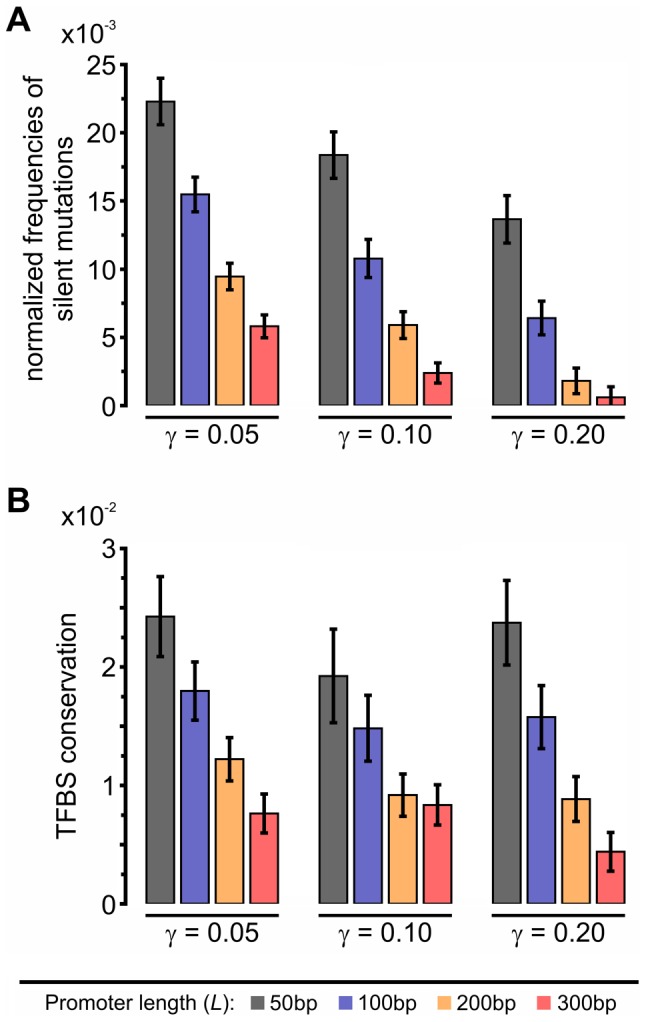
Local-sequence level mechanisms. (A) Resistance to creation of TFBSs within TFBS-free regions, measured as the frequency of silent mutation events (Fig. 4) normalized to the fraction of TFBS-free region in the genome. (B) TFBS conservation or degree of resilience to deletion of TFBSs, measured as the average probability that a TFBS will remain a TFBS following a point mutation. Error bars are computed as the standard error of the mean over 100 independent simulations.

By applying single point mutations to an individual (see Materials and Methods) we can record the frequency of occurrence of each type of mutation as well as their average effect on the phenotype. We observe that that frequency of all mutation types is gradually decreasing with increasing specificity gap (*γ*) and URR lengths (*L*) (Fig. 5A). Among all the mutation types, under all conditions, the silent mutations dominate (Fig. 5A). This is especially true at low specificity gap (*γ*) and URR lengths (*L*). Since silent mutations do not affect the phenotype, we hypothesized that they could be indirectly involved in the gain of robustness through an unknown mechanism. The frequency of silent mutations could increase for two reasons. First, the odds of a silent mutation may increase with increasing TFBS-free promoter regions, and second, TFBS-free regions could become more resilient to the creation of new TFBSs. These two possibilities are intertwined as a result of the dramatic drop in connectivity observed at lower values of *γ* (Fig. 3B). However, the latter one can be estimated by dividing the frequency of silent mutations with the size of TFBS-free region, at each time point during the simulation. Fig. 6A shows that TFBS-free promoter regions indeed evolve sequences that are more resilient to the creation of new TFBSs upon mutations, a mechanism that we term “*TFBS avoidance”*. A further verification of this effect is the observed decreased probability of generating a TFBS in the TFBS-free regions upon point mutations (Fig. S6). This decrease is larger for sparser networks, correlating well with the normalized frequencies of silent mutations shown in Fig. 6A. Next, we estimated the relative contribution of TFBS avoidance to the increase in robustness (see Materials and Methods) (Fig. 5B). Clearly, the local-sequence contribution is more important at low values of *γ* as well as at low URR lengths (*L*).

Another possible mechanism underlying the increase in robustness is related to changes in the “preserved” binding events (Fig. 4A). Robustness should increase proportionally to the degree to which a TFBS is preserved in the face of point mutations, a property we call “*TFBS conservation*”. We found that, generally speaking, the more similar a TFBS is to the consensus one (i.e. more conserved), the less likely a single mutation will make it a non-binder. We cannot capture this phenomenon directly from frequency values due to the confounding effects of decreasing connectivity. Therefore, we quantify TFBS conservation as the fraction of single point mutants (there are 8×3 = 24 such mutant sequences for any given 8-mer) that remain as binding sites for the same TF (Fig. 6B). A binding site with greater TFBS conservation is more resilient to point mutations and should therefore contribute to greater robustness. Because in our structural model the atomic contacts at the interface between the TF and the DNA molecules are explicitly modeled, our model should capture the complexity of the set of binding sites [22] including any TFBS conservation features. The average TFBS conservation (Fig. 6B) follows the same behavior as the TFBS avoidance (Figs. 6A and S6), but its contribution to robustness is limited, since for example only 12% of the robustness remains unexplained in the case of *γ* = 0.05 and *L* = 50 bp (Fig. 5B, brown portion of the bar) where TFBS conservation is at a maximum (Fig. 6B). This number, 12%, thus represents an upper bound for the contribution of the TFBS conservation mechanism to robustness. The similar behavior observed for TFBS conservation and TFBS avoidance suggests that the former mechanism could be conceptually classified more as a local-sequence level mechanism.

### Network-architecture level mechanisms buffer the detrimental effect of mutations at longer promoter lengths

The remaining mechanisms to be analyzed are related to the effect of redundant sites and unique sites (Fig. 3A). Redundant sites can reinforce network connections, conferring robustness to the system, while the creation and deletion of unique sites rewires the genetic network, giving an opportunity to explore different qualitative connections and select those that more effectively buffer the effect of mutations.

To better understand the role of redundancy we measured the net amount of redundant TFBSs at the end of the simulations. There is a trivial increase in redundancy associated with increased network connectivity (Fig. S7A). Therefore, we corrected this effect by subtracting the amount of redundant sites that emerged in randomly generated networks with similar connectivity (Fig. S7B). We find that the net gain in redundancy is small or nonexistent, meaning that redundancy is constantly maintained during the simulations. The constant redundancy is reflected in the “uncorrected redundancy” (Fig. S7C). We observe that for longer URRs their values are quite large. At first, this seems to suggest that robustness should be large at long URRs, but if this were the case, then it should be large even at the start of the simulations, which it is not what we observe. Instead, networks with high redundancy display the smallest values of initial robustness (Fig. S3). This suggests that if redundancy is being used by highly interconnected systems to increase robustness, it may be done by relocating redundant inputs and reinforcing genetic interactions that are important in determining the phenotype of the individuals.

**Figure 7:**
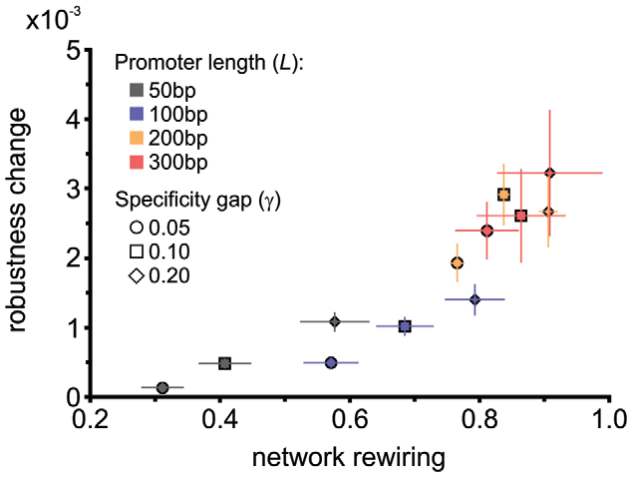
Network-architecture level mechanisms. Correlation between the “other contributions” portion of the change in robustness (Fig. 5B) and the average network rewiring as a function of URR (*L*) and specificity gap (γ). Rewiring, *Φ*, was computed between individuals at generation 2000 and their respective founders (see Materials and Methods). These values were corrected for the effects of changing connectivity by calculating *Φ* between two randomly chosen stable individuals, both with the same average connectivity values observed for the individuals at the end of the simulations. The correlation shows that *Φ* explains for the most part the “other contributions” component of robustness. The amount of rewiring depends primarily on *L* and to a lesser extent on *γ*. Error bars are the standard error of the mean over 100 independent simulations.

Now, consider the role of creating and deleting unique binding sites. By definition, these events rewire the network, introducing changes at the network-architecture level (Fig. 4A). We can calculate the contribution of network rewiring (*Φ*) by measuring the normalized amount of network changes with respect to the Founder in each simulation (see Materials and Methods). *Φ* is a number between 0 and 1, where 1 means that all the connections changed, giving the maximum network rewiring. Fig. 7 shows the correlation between the “other contributions” to robustness (brown portions of the bars in Fig. 5B) and *Φ*. An overall exponential correlation can be observed, which explains the increase in robustness. According to Fig. 7, *Φ* depends mainly on the length of the promoter region and to a much lesser degree on the specificity gap (*γ*). *γ* affects TF expression levels, which are controlled by a sigmoid curve, therefore large values tend to saturate TF expression levels, favoring turnover of binding sites by making changes neutral (same TF expression value irrespective of the changing *κ*).

Network rewiring explains the larger robustness changes and shows that it is an effective mechanism for the creation of robustness. In summary, as the networks evolve, TFBSs are rearranged, thus creating and deleting network connections and occasionally forming, with the opportunity to fix, specific network motifs (including reinforced connections due to the existence of TFBS redundancy) that have an improved capacity to absorb the detrimental effects of the introduced mutations.

## Discussion

The available structural information about TF-DNA complexes allowed us to construct an explicit DNA sequence based model of gene regulatory networks for the study of the genetic mechanisms that make a biological system robust to mutations. The level of connectivity and architecture of gene regulatory networks in biological systems is still being explored, therefore in this study we considered and discussed networks that span a range of interconnectedness between genes. While our model has limitations, most notably the simplified model of transcriptional regulation where no restrictions are applied to positioning or interaction between TFBSs, it has allowed us to probe the mechanisms underlying robustness in gene networks at the DNA level. By extending this approach in the future, more detailed questions will become amenable to investigation, for example, understanding the evolutionary forces that shape promoter architectural features such as TFBS clustering and cooperativity. However, addressing questions such as these will require more detailed modeling of the transcriptional process, perhaps by incorporating thermodynamics-based models that include distance-dependent interactions among TFBSs and position dependent negative regulation or repression [28]. The model could also incorporate more complicated population-level mechanisms, such as recombination within promoter regions, as it has been shown to evolve robustness even more efficiently [9].

Our results showed that sparse networks, which could be broadly related to gene regulatory mechanisms in simpler organisms, show a contribution from both local-sequence level and network-architecture level mechanisms to robustness. These sparse networks have difficulties in rewiring connections, since the few available need to be conserved to maintain a viable phenotype. Instead, these networks rely on local-sequence mechanisms, manipulating the genetic sequence to decrease the probability of generating spurious binding sites *de novo* (TFBS avoidance mechanism) and decrease the probability of losing existing binding sites (TFBS conservation mechanism). When assuming a small specificity gap between non-specific and specific binding (*γ*), TFBS avoidance becomes a dominant driving force in evolving robustness. On the other hand, when greater robustness in sparse networks is achieved by means of a larger specificity gap, it happens by balancing out TFBS avoidance, TFBS conservation and network rewiring. Meanwhile, in more interconnected networks, which one could relate to higher level organisms, we observe a complete shift towards the use of network-architecture level mechanisms. Specifically, these networks rewire, exploring different network motifs and fixing those that dampen the harmful effects of mutations. We would argue that this rewiring constitutes an increase in complexity of the network. Evolution of network complexity in more interconnected networks thus appears as the dominant source of robustness, especially in networks with a large specificity gap separating binding and non-binding sites.

There is evidence in the literature of the existence of all three observed mechanisms. i) TFBS avoidance: Hahn and coworkers examined polymerase binding regions in Eubacteria and Archaea genomes and showed that polymerase binding sites were under-represented in binding-free regions, even more so in Eubacteria [52]. ii) TFBS conservation: Wunderlich and Mirny [53] compared the information content of TFBSs in prokaryotes and eukaryotes, finding that the simpler prokaryotic organisms use TFBSs with higher information content than the more complex eukaryotic organisms. Higher information content is equivalent to higher TFBS conservation values in our simulations, since both measures are directly proportional to the interaction strengths of TFBSs (see Fig. S8 for the relationship with our calculated binding specificities). iii) Network complexity: the use of this mechanism requires extensive turnover of TFBSs and there is evidence in the literature of this phenomenon. For example, Bradley et al. observed that the same genes in closely related species of Drosophila were differently regulated [54] and Ben-Tabou de-Leon and Davidson also showed differences at the promoter level in gene regulatory networks of two related species, the sea star and sea urchin [55]. The existence of network motifs that are robust to interferences have also been previously described [56], [57], but the conditions of their evolution still remains under scrutiny [58].

Depending on the explored parameters in our simulations, we observed a varied interplay of the different mechanisms. While it is uncertain which of them better reflects real biological organisms, it is encouraging that our model demonstrated all the three naturally observed mechanisms. Leclerc [13] previously estimated the number of TF inputs per gene in gene regulatory networks for different organisms and found them to be sparsely connected, with values ranging from 1.37 (*Escherichia coli*) to 2.75 (*Arabidopsis thaliana*). However, this analysis did not contain measurements for higher organisms such as mouse or human, for which the number of inputs per gene could be higher given the apparent trend of increasing number of TF inputs per gene with organismal “complexity”. According to these estimates, promoters with more than 100 bp in our linear model would result in unrealistic levels of interconnections.

The evolution of increased complexity is a major unaddressed question in Biology [59]. Our results suggest a potential path for increased complexity as a consequence of the shift to network-architecture level changes when more interconnected networks are considered. Two outcomes suggest that network-architecture level changes are more effective at evolving robustness than local-sequence changes. First, local-sequence level changes were not used under high connectivity in spite of also having equal, if not greater, access to these types of changes. This suggests that robustness at the network level is more easily evolved. Second, the increase in robustness is stronger under conditions of high connectivity (Fig. 3A). Thus, high connectivity settings create favorable conditions to employ more effective network-architecture level changes towards evolving robustness.

## Materials and Methods

### Modeling of TF-DNA complexes

10 TF-DNA complexes with resolution below 2.3 Å were selected from the Protein Data Bank (www.pdb.org) such that the TF was in contact with 8 or 9 bases on the DNA. DNA structures of length 9 were shortened to length 8 by discarding the terminal base with the least number of atomic contacts. We modeled TF-DNA complexes with all possible 4^8^ (65536) different DNA segments of length 8 (8-mers) as follows. The original nucleic acid bases were stripped in the coordinates file and replaced by those corresponding to the desired sequence (all but the bases' atom triad of one nitrogen and two carbons attached to the sugar, which retain information about the planarity of the base) using the program psfgen from NAMD 2.6. TF structures were not changed. To optimize the interactions between interfacing atoms, the resulting TF-DNA complexes were minimized for 3000 steps using the conjugate gradient algorithm in NAMD 2.6 with the CHARMM force field. The simulation took place in vacuum, retaining any available crystal water molecules.

### TF-DNA binding specificity, *κ*

We used an all-atom, distance-dependent statistical pair potential [34] to obtain normalized statistical preferences (*ε′*) of the 10 chosen transcription factors bound to each of the 4^8^ (65536) possible 8-mers. The function considers protein and nucleic acid heavy atoms in a residue-specific manner and maps the continuous value of distances *d_ij_*, between atoms *i* and *j*, to a set of distance bins, only counting atoms falling in the *d_ij_* range. We used the following function parameters: *i)* a maximum distance of 10 Å, considered between any two interface atoms, *ii)* a 3 Å distance for the first bin and *iii)* 1 Å distance for the remaining 7 bins, giving a total of 8 distance bins. In order to score the set of TF-DNA complexes for a given transcription factor we trained the statistical potential on the original TF-DNA crystal complex, which we found gave a better performance than the standard approach of using several TF-DNA crystal complexes.

### Gene expression dynamics

Equation IV is applied iteratively until either reaching a steady state 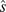, or a time limit. The steady state 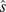 is defined when a measure 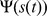 analogous to a variance over the last 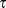 time-steps is less than 10^−4^:
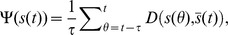
where 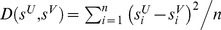 is a distance metric between two expression vectors of length *n* and 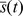 represents the mean gene expression across the interval 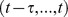. If the system does not reach equilibrium before 100 iterations, it is considered unstable. The phenotype of each individual is defined by the final stable equilibrium expression pattern, denoted 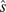. For all simulations presented, sigmoid slope *α* = 20, the number of genes *n* = 10 and *τ* = 10.

### Selection

At each generation, offspring are selected based upon their fitness. For each individual we compute their phenotypic similarity to the founder, which is measured as the mean distance to the optimal phenotype, 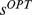. Then, we define fitness as:
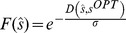
where *σ* modulates the strength of selection. Our results use strong stabilizing selection, i.e. *σ* = 0.001. Offspring that are not viable (i.e., no stable equilibrium found) are assigned fitness zero.

### Robustness to mutations

Each individual in the population is subjected to 100 independent random single point mutations. Following this, each mutated individual undergoes development (we discard unstable solutions) and the phenotypic distance, *D*, between the mutated individual and the original unmutated individual is measured (see “Gene expression dynamics” above for definition of *D*). Then, the robustness of an individual is defined as the average phenotypic distance over the 100 perturbations.

### Local-sequence level contribution to robustness to mutations

By applying single point mutations to an individual we can record the frequency of occurrence of each type of mutation as well as the average effect they have on the phenotype. In this manner, the observed robustness becomes the product of the average phenotypic effect of a mutation *i* (*e_i_*) and the frequency at which it occurs (*f_i_*). The frequencies (only from stable individuals) at any given generation add up to 1, therefore the sum of the changes in frequencies between the beginning and end of the simulation for each mutation type is zero:
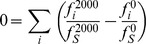

*f_i_^2000^* and *f_i_^0^* are the frequencies at generations 2000 and 0 for a given mutation type *i*, and *f_S_^2000^* and *f_S_^0^* are the total frequencies of the stable individuals at generations 2000 and 0, used to normalize for changes in the amount of stable individuals. This equation is used to generate Fig. 5A.

We estimate the contribution of silent mutations to robustness by assuming that the negative frequency changes (mutations that lower the frequency over time in response to the increasing frequency of silent mutations) remain constant for the duration of the simulation and compare them to the observed robustness:
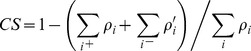
CS is the fractional contribution of silent mutations to the change in robustness (discussed and shown in Fig. 5B of the main text). Mutations with positive indices (*i^+^*) indicate the sum over mutation events whose changes in frequencies are positive and mutations with negative indices (*i^−^*) similarly indicate the sum over those with negative frequency changes. *ρ_i_* is the observed robustness change and *ρ′_i_* is the robustness change assuming constant frequency, detailed as follows:
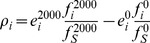


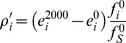
where *e_i_^2000^* and *e_i_^0^* are the average phenotypic distances at generations 2000 and 0 for each type of mutations. Note that in the robustness change at constant frequency (*ρ′_i_*) the ratio 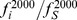 equals that of the initial generation (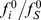).

### Estimation of network rewiring

We estimate network rewiring by measuring the number of changes (gene connections gained and lost) of individuals in the evolving population with respect to the founder. We use a normalized form of network rewiring, *Φ*, expressed as follows:
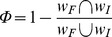

*Φ* is a number between 0 and 1, where 1 represents the maximum network change or rewiring; *w_F_* and *w_I_* are the sets of nonzero entries in the matrices of respectively the founder *F* and a given individual *I* in the population. The equation calculates the complement of the normalized number of common interactions (intersection between matrix elements), which quantifies the number of changes in the *w* matrices. As the promoter length increases, it becomes easier to observe common interactions that happen by chance. Consequently, we measured *Φ* for randomly generated networks and found a linear correlation with network connectivity (Fig. S9). We correct *Φ* for spurious common connections using the regression equation as follows:
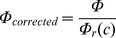
where 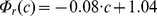 and is the network rewiring from random networks. c represents the network connectivity.

## Supporting Information

Figure S1 **Statistical pair potential scores,** ***ε*** **, scale linearly with the number of TF-DNA atomic contacts.** We collected 162 TF-DNA complexes from the Protein Data Bank and measured the number of TF-DNA atomic contacts (using a cutoff value of 5 Angstroms) and plotted them against their TF-DNA interaction strengths, *ε_x_* (*x* is the DNA sequence in the crystal). Only non-hydrogen atoms were considered. We observe that these two measures are correlated (r^2^ = 0.47 -fit is shown as a green line). Therefore, in order to compare scores from two different TF-DNA complexes, it is necessary to apply a transformation and obtain normalized scores in the range from 0 to 1.
(TIF)

Figure S2 **Phenotypic distance of unstable individuals.** Unstable individuals are spontaneously generated when mutations are introduced to measure robustness via phenotypic distance between perturbed and unperturbed individuals. Here, we show average phenotypic distances of unstable individuals throughout the simulations for different parameters, promoter length (*L*) and specificity gap (*γ*). The contribution to robustness due to unstable individuals was discarded on the basis of their quasi-random values across each simulation and their very low frequency of occurrence (∼2%). Error bars are the standard error of the mean over 100 independent simulations.
(TIF)

Figure S3 **Evolution of robustness as a function of specificity gap and promoter length.** Robustness was measured as the phenotypic distance between perturbed and unperturbed individuals as a function of time, in generations using a logarithmic scale. Each point represents the average phenotypic distance over 100 independent simulations (standard error of the mean is displayed). The upper panel illustrates that robustness reaches a maximum in each simulation. The statistical significance of the differences of robustness values over the length of simulation with respect to generation 2000 were calculated using t-test. The p-values are displayed in the lower panel. The differences become insignificant approximately after generation 500 in all cases.
(TIF)

Figure S4 **Connectivity and number of TFBSs as a function of the** ***cis*** **-regulatory region length (** ***L*** **).** The number of TFBSs displays a linear relationship with *L* (blue curve). On the other hand, network connectivity, measured as the sum of the number of unique inputs on each gene in a given network expressed as a fraction of the total, shows a saturating curve (grey curve). Network connectivity saturates at high promoter lengths because there is a limited amount of transcription factors in the system. The difference between the two curves represents the initial random amount of redundant inputs at given values of *L*. Error bars are standard deviations of 500 randomly generated networks for which we measured both connectivity and number of TFBSs.
(TIF)

Figure S5 **Internal composition of the “combined” category of mutations.** Frequencies of mutational categories as described in Fig. 4A (left plots). On the right plots we display the relative frequencies of the individual components within the “combined” category. The internal composition of the combined events (right plots) reflects the frequencies of the individual categories (left plots). Error bars are the standard error of the mean over 100 independent simulations.
(TIF)

Figure S6 **Propensity of generating TFBSs** ***de novo*** **in TFBS-free promoter regions.** For each 8-mer in the TFBS-free regions of the promoters we computed the fraction of single point mutations that turns an 8-mer into a TFBS for any TF, which represents the probability of generating a TFBS *de novo* upon a point mutation. We show here the average propensity of all 8-mers in TFBS-free regions as a function of promoter length (*L*) and specificity gap (*γ*). We observe an increased resistance to the creation of TFBSs for small *L*'s and *γ*'s. Error bars are the standard error of the mean over 100 independent simulations.
(TIF)

Figure S7 **The use of redundancy of TFBSs.** (A) Relationship between redundant sites and network connectivity (also proportional to promoter length -Fig. S4). There is a strong correlation between the two observables, showing TFBS redundancy (as derived from randomly generated networks) as a function of the conditions of the simulation. (B) Net redundancy, computed from TFBS redundancy of individuals at generation 2000, corrected by subtracting TFBS redundancy calculated from random networks that used the same average network connectivity as the measured individuals. (C) Uncorrected TFBS redundancy for individuals at generation 2000. Error bars are the standard error of the mean of 100 independent simulations.
(TIF)

Figure S8 **Relationship between TFBS conservation and binding specificity.** The plots compare the degree of TFBS conservation (see main text for definition) with the calculated binding specificities for each TF. The table at the bottom shows correlation coefficients for each of the scatter plots. TFBS conservation and TFBS specificities are highly correlated.
(TIF)

Figure S9 **Spurious network rewiring as a function of network connectivity.** We measured the amount of spurious network rewiring for different URR lengths and found both measures linearly correlated within the tested range. Spurious rewiring decreases due to an increase in the probability of finding common connections between two different networks as a function of network connectivity. The green solid line corresponds to the linear fit (equation and R^2^ correlation are also displayed). Error bars are the standard deviation on each measure computed from 500 randomly generated network pairs.
(TIF)

Table S1 **List of transcription factors used in the simulations.** List of 10 TF-DNA complexes available in the Protein Data Bank [37] that were used for network simulations. The provided number of TFBS describe those 8-mers displaying relative binding scores (*ε′*) greater than *ε′_opt_* = 0.209, which produced the expected number of TFBSs closest to the average in the range of 60–900 for all considered TFs.
(DOC)
