# Supplementary figures and images for: The Underlying Molecular and Network Level Mechanisms in the Evolution of Robustness in Gene Regulatory Networks
Source: PLoS Comput Biol. 2013 Jan 3;9(1):e1002865. doi: 10.1371/journal.pcbi.1002865 (PMC3536627; doi:10.1371/journal.pcbi.1002865)

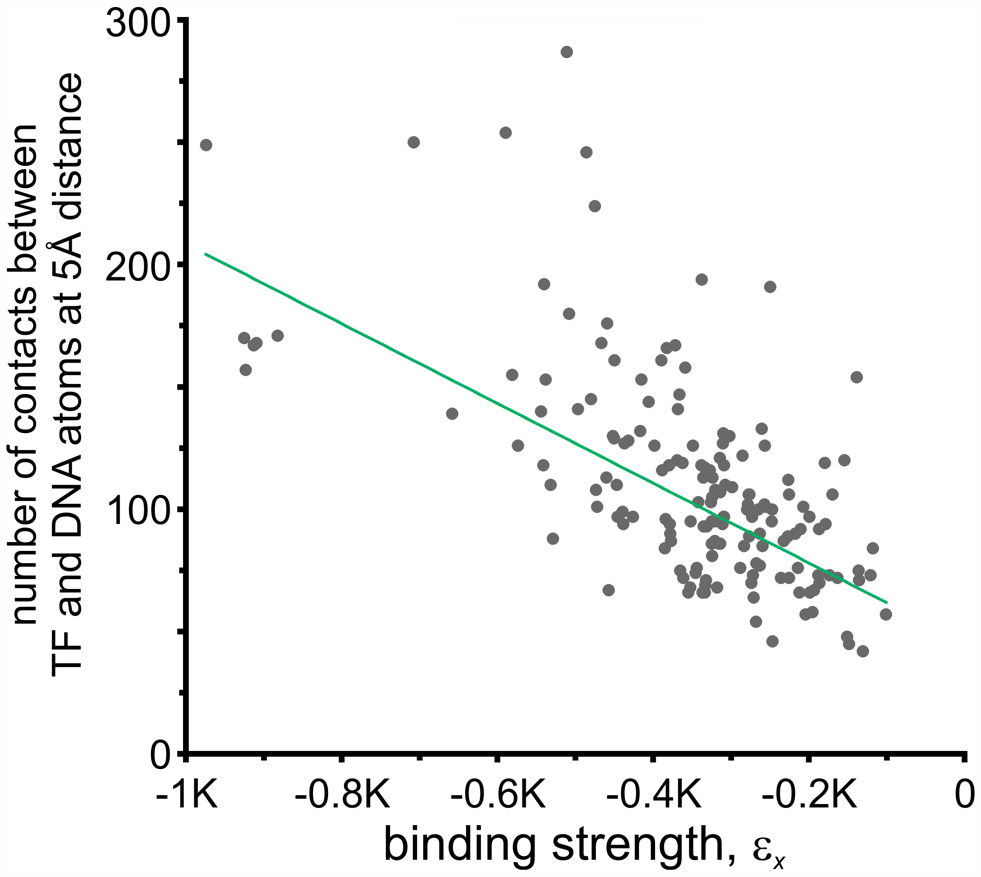

Supplement: Figure S1 — Statistical pair potential scores, ε , scale linearly with the number of TF-DNA atomic contacts. We collected 162 TF-DNA complexes from the Protein Data Bank and measured the number of TF-DNA atomic contacts (using a cutoff value of 5 Angstroms) and plotted them against their TF-DNA interaction strengths, εx (x is the DNA sequence in the crystal). Only non-hydrogen atoms were considered. We observe that these two measures are correlated (r2 = 0.47 -fit is shown as a green line). Therefore, in order to compare scores from two different TF-DNA complexes, it is necessary to apply a transformation and obtain normalized scores in the range from 0 to 1. (TIF) [file pcbi.1002865.s001.tif]

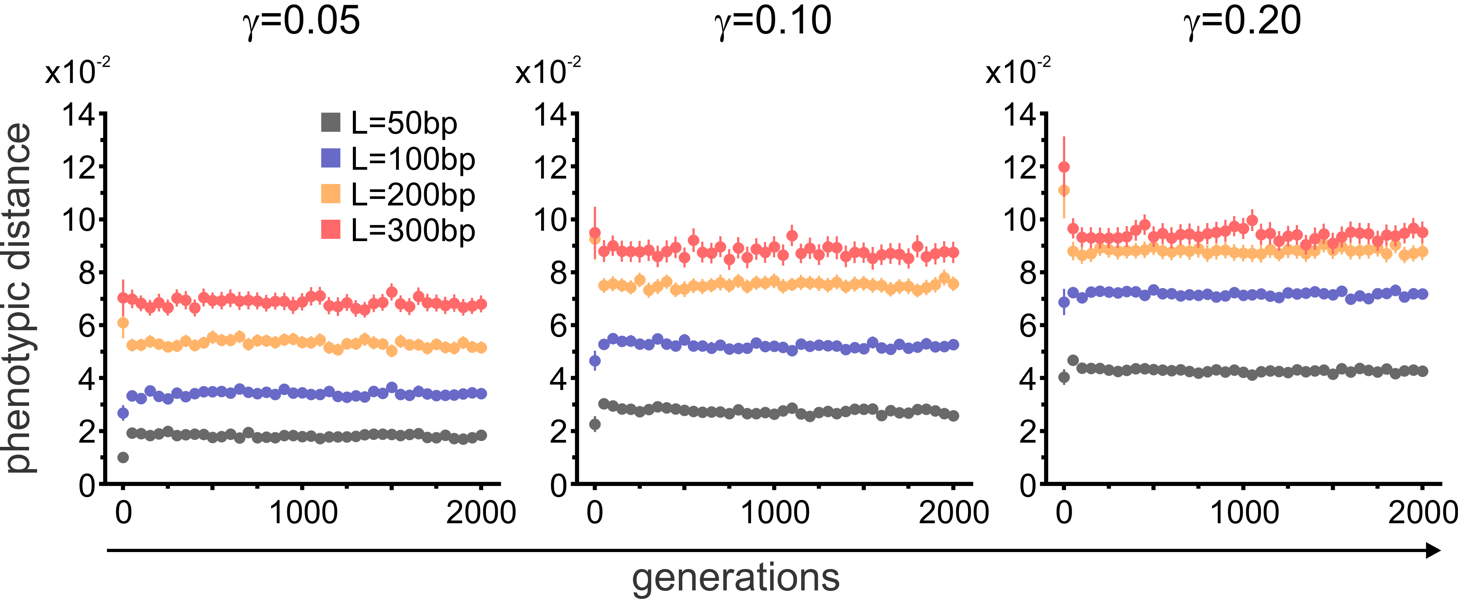

Supplement: Figure S2 — Phenotypic distance of unstable individuals. Unstable individuals are spontaneously generated when mutations are introduced to measure robustness via phenotypic distance between perturbed and unperturbed individuals. Here, we show average phenotypic distances of unstable individuals throughout the simulations for different parameters, promoter length (L) and specificity gap (γ). The contribution to robustness due to unstable individuals was discarded on the basis of their quasi-random values across each simulation and their very low frequency of occurrence (∼2%). Error bars are the standard error of the mean over 100 independent simulations. (TIF) [file pcbi.1002865.s002.tif]

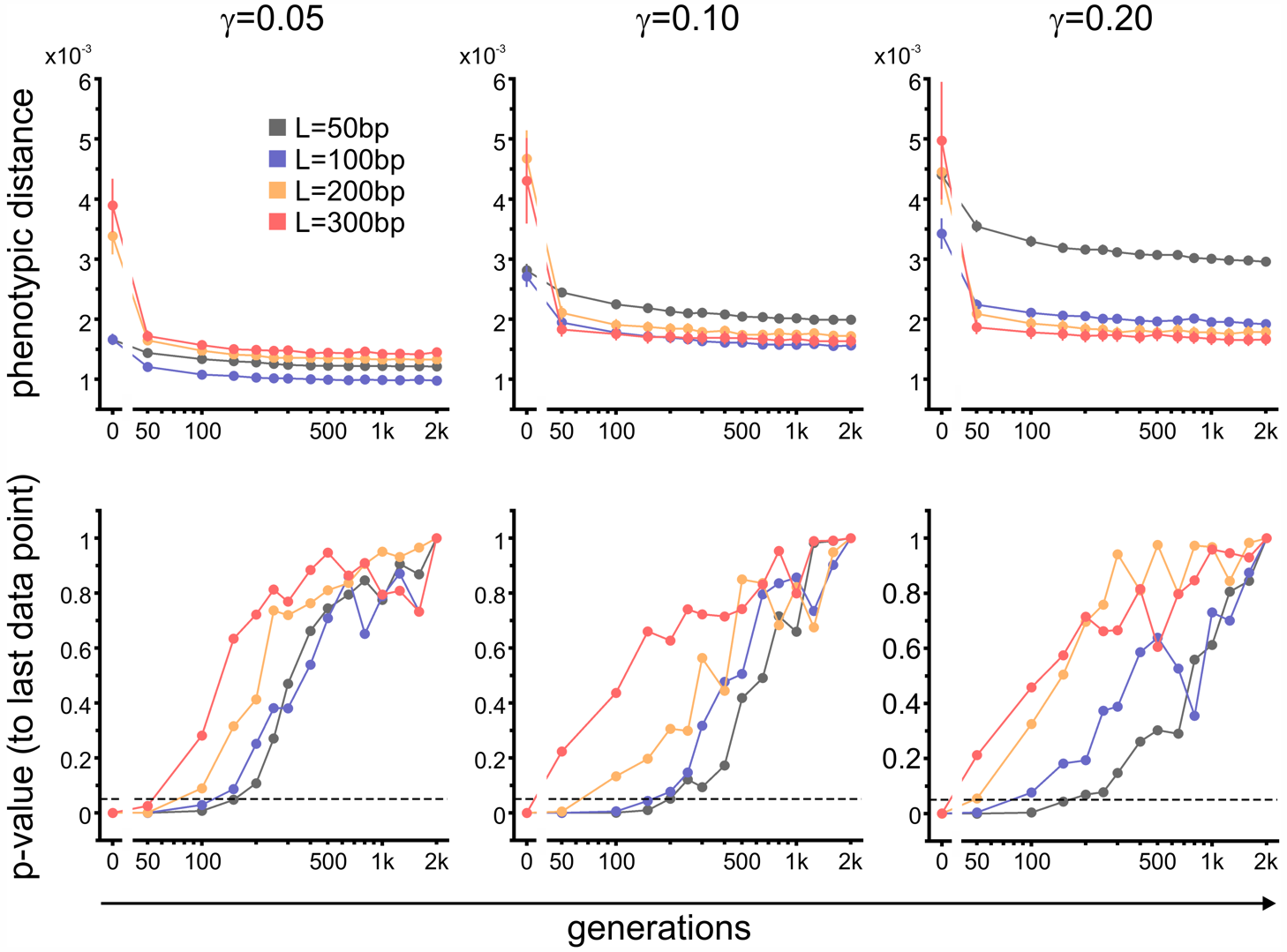

Supplement: Figure S3 — Evolution of robustness as a function of specificity gap and promoter length. Robustness was measured as the phenotypic distance between perturbed and unperturbed individuals as a function of time, in generations using a logarithmic scale. Each point represents the average phenotypic distance over 100 independent simulations (standard error of the mean is displayed). The upper panel illustrates that robustness reaches a maximum in each simulation. The statistical significance of the differences of robustness values over the length of simulation with respect to generation 2000 were calculated using t-test. The p-values are displayed in the lower panel. The differences become insignificant approximately after generation 500 in all cases. (TIF) [file pcbi.1002865.s003.tif]

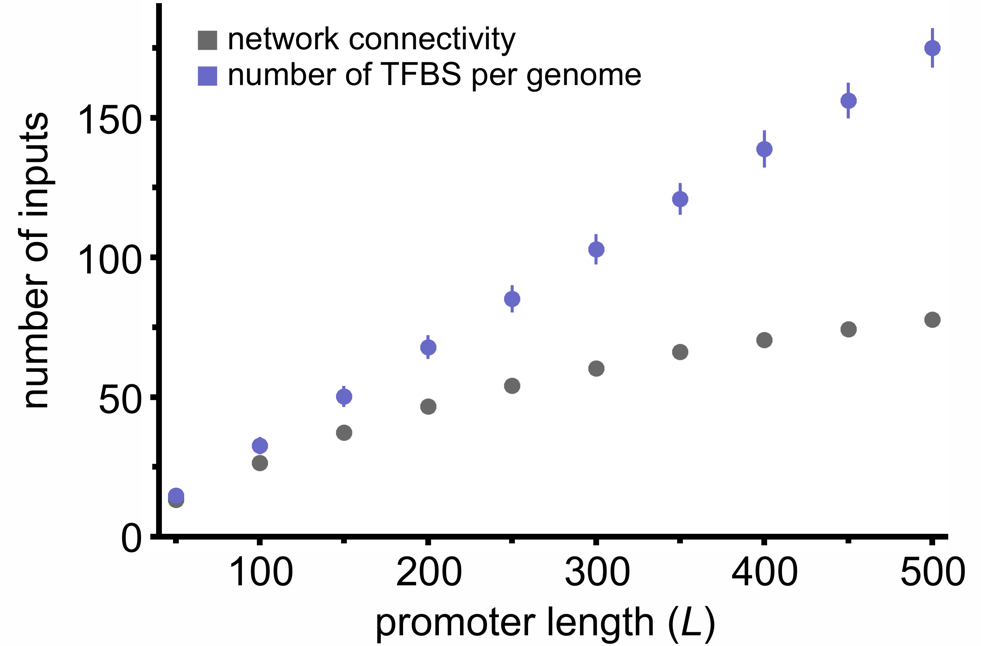

Supplement: Figure S4 — Connectivity and number of TFBSs as a function of the cis -regulatory region length ( L ). The number of TFBSs displays a linear relationship with L (blue curve). On the other hand, network connectivity, measured as the sum of the number of unique inputs on each gene in a given network expressed as a fraction of the total, shows a saturating curve (grey curve). Network connectivity saturates at high promoter lengths because there is a limited amount of transcription factors in the system. The difference between the two curves represents the initial random amount of redundant inputs at given values of L. Error bars are standard deviations of 500 randomly generated networks for which we measured both connectivity and number of TFBSs. (TIF) [file pcbi.1002865.s004.tif]

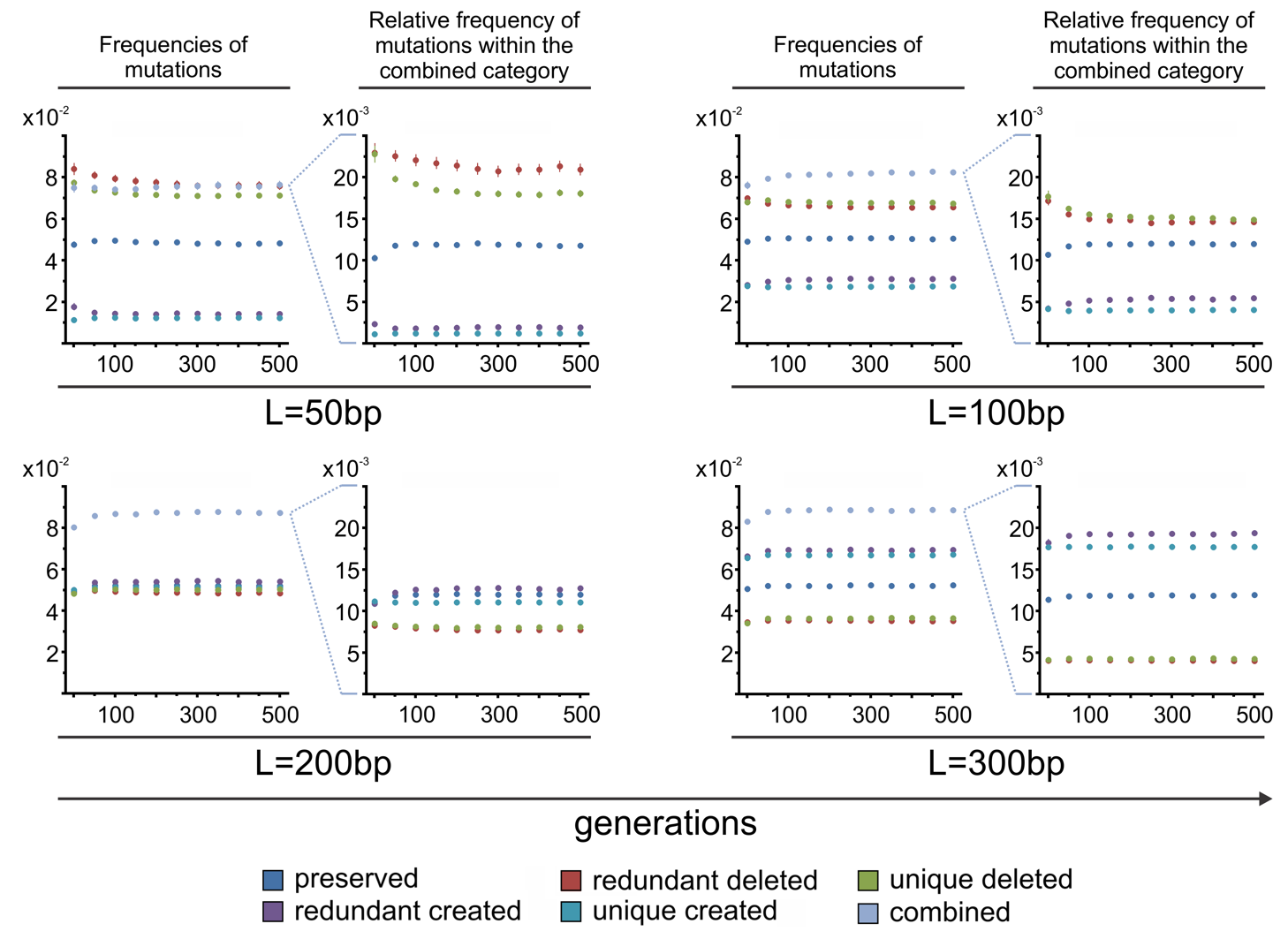

Supplement: Figure S5 — Internal composition of the “combined” category of mutations. Frequencies of mutational categories as described in Fig. 4A (left plots). On the right plots we display the relative frequencies of the individual components within the “combined” category. The internal composition of the combined events (right plots) reflects the frequencies of the individual categories (left plots). Error bars are the standard error of the mean over 100 independent simulations. (TIF) [file pcbi.1002865.s005.tif]

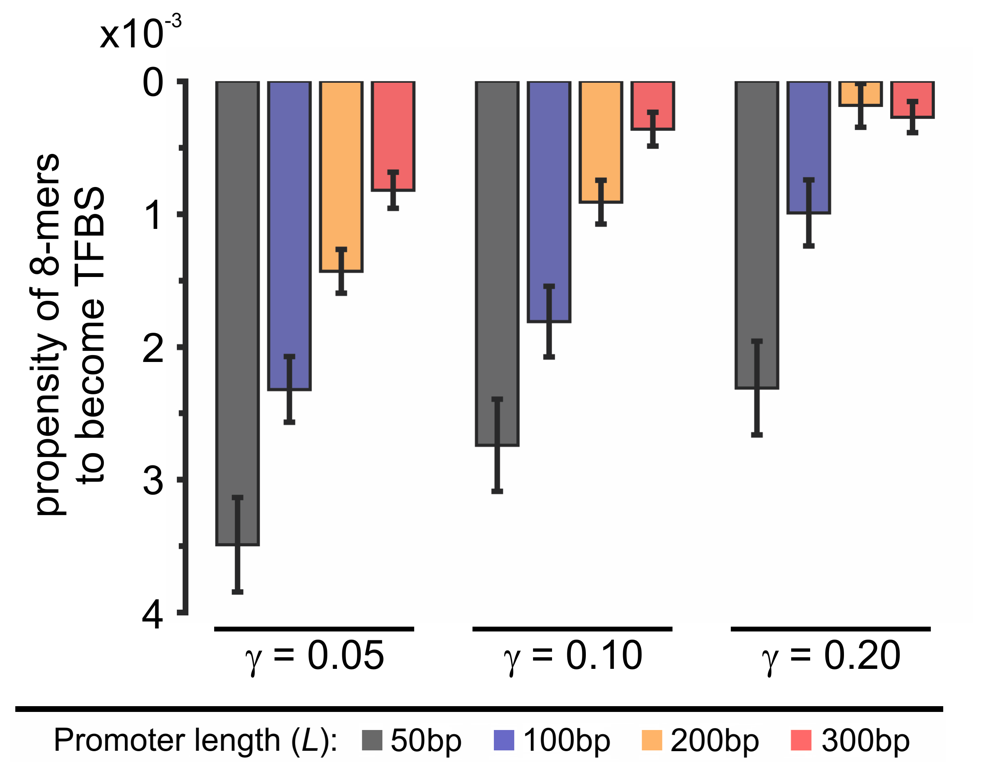

Supplement: Figure S6 — Propensity of generating TFBSs de novo in TFBS-free promoter regions. For each 8-mer in the TFBS-free regions of the promoters we computed the fraction of single point mutations that turns an 8-mer into a TFBS for any TF, which represents the probability of generating a TFBS de novo upon a point mutation. We show here the average propensity of all 8-mers in TFBS-free regions as a function of promoter length (L) and specificity gap (γ). We observe an increased resistance to the creation of TFBSs for small L's and γ's. Error bars are the standard error of the mean over 100 independent simulations. (TIF) [file pcbi.1002865.s006.tif]

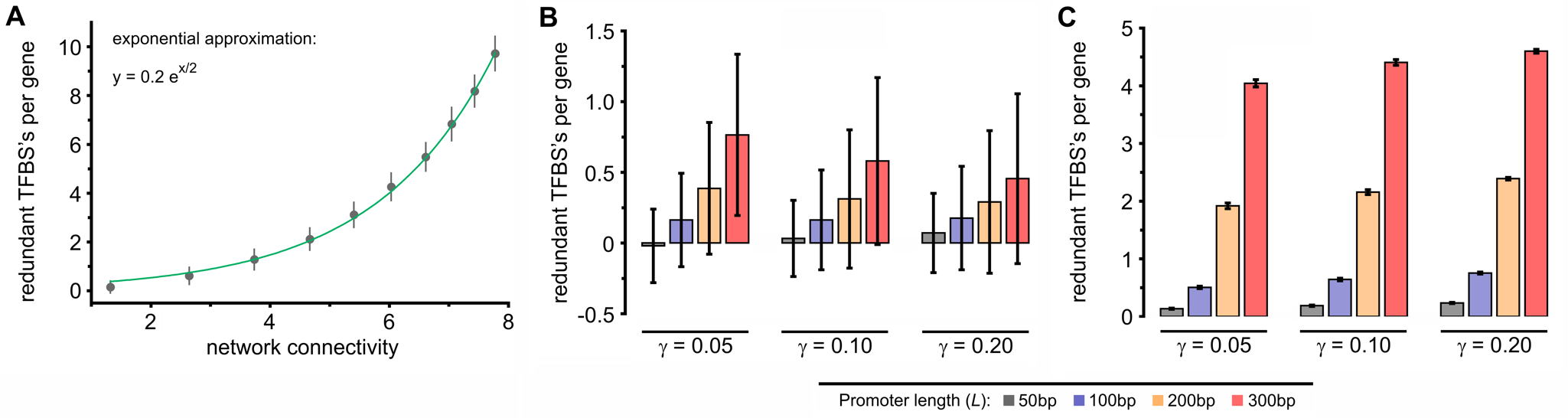

Supplement: Figure S7 — The use of redundancy of TFBSs. (A) Relationship between redundant sites and network connectivity (also proportional to promoter length -Fig. S4). There is a strong correlation between the two observables, showing TFBS redundancy (as derived from randomly generated networks) as a function of the conditions of the simulation. (B) Net redundancy, computed from TFBS redundancy of individuals at generation 2000, corrected by subtracting TFBS redundancy calculated from random networks that used the same average network connectivity as the measured individuals. (C) Uncorrected TFBS redundancy for individuals at generation 2000. Error bars are the standard error of the mean of 100 independent simulations. (TIF) [file pcbi.1002865.s007.tif]

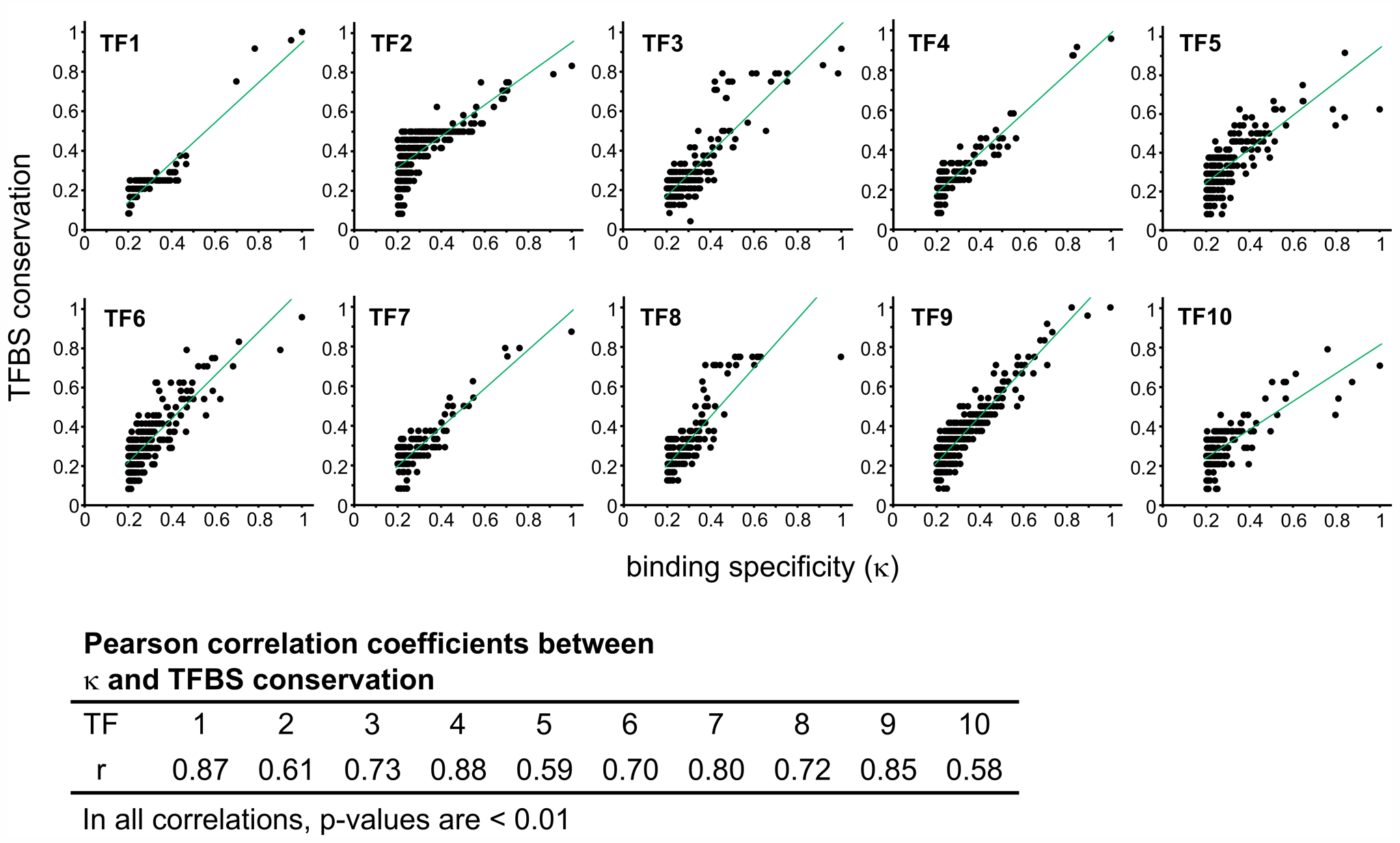

Supplement: Figure S8 — Relationship between TFBS conservation and binding specificity. The plots compare the degree of TFBS conservation (see main text for definition) with the calculated binding specificities for each TF. The table at the bottom shows correlation coefficients for each of the scatter plots. TFBS conservation and TFBS specificities are highly correlated. (TIF) [file pcbi.1002865.s008.tif]

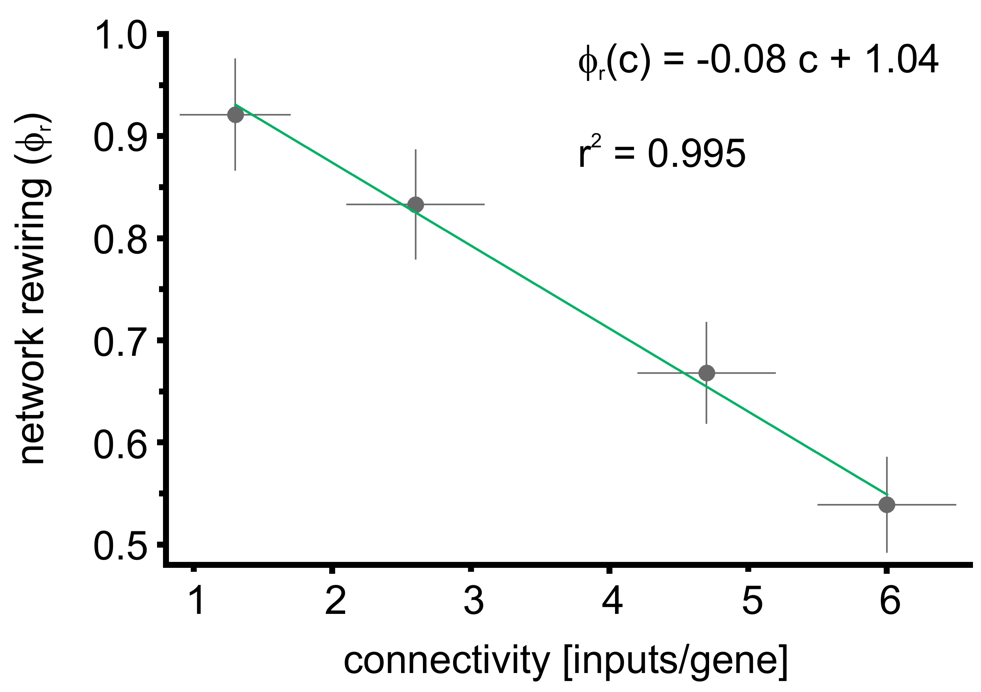

Supplement: Figure S9 — Spurious network rewiring as a function of network connectivity. We measured the amount of spurious network rewiring for different URR lengths and found both measures linearly correlated within the tested range. Spurious rewiring decreases due to an increase in the probability of finding common connections between two different networks as a function of network connectivity. The green solid line corresponds to the linear fit (equation and R2 correlation are also displayed). Error bars are the standard deviation on each measure computed from 500 randomly generated network pairs. (TIF) [file pcbi.1002865.s009.tif]
