# Supplementary material for: The Underlying Molecular and Network Level Mechanisms in the Evolution of Robustness in Gene Regulatory Networks
Source: PLoS Comput Biol. 2013 Jan 3;9(1):e1002865. doi: 10.1371/journal.pcbi.1002865 (PMC3536627; doi:10.1371/journal.pcbi.1002865)
Supplement: Table S1 — List of transcription factors used in the simulations. List of 10 TF-DNA complexes available in the Protein Data Bank [37] that were used for network simulations. The provided number of TFBS describe those 8-mers displaying relative binding scores (ε′) greater than ε′opt = 0.209, which produced the expected number of TFBSs closest to the average in the range of 60–900 for all considered TFs. (DOC) [file pcbi.1002865.s010.doc]

| **TF#** | **PDB code** | **Organism** | **TF Name** | **Number of TFBS** |
| --- | --- | --- | --- | --- |
| 1 | 1AAY | *Mus musculus* | Zif268 or Egr1 (Early Growth Response protein 1) | 95 |
| 2 | 1B72 | *Homo sapiens* | HoxB1 (Homeobox B1) | 326 |
| 3 | 1BC8 | *Homo sapiens* | Elk4 or SAP-1 (ETS family of transcription factors) | 244 |
| 4 | 1BY4 | *Homo sapiens* | Rxr-A (retinoid X receptor, alpha) | 153 |
| 5 | 1CQT | *Homo sapiens* | OCA-B or OBF-1 or BOB-1 | 248 |
| 6 | 1E3O | *Homo sapiens* | POU homeobox protein Oct-1 | 298 |
| 7 | 1K78 | *Homo sapiens* | Paired box protein PAX5 | 136 |
| 8 | 1PUF | *Mus musculus* | HoxA9 (homeobox protein A9) | 232 |
| 9 | 1QN4 | *Arabidopsis thaliana* | TBP (transcription initiation factor TFIID-1) | 424 |
| 10 | 1T2K | *Homo sapiens* | IRF3 (interferon regulatory factor 3) | 159 |
